# The neurovascular unit and its correlation with cognitive performance in patients with cerebral small vessel disease: a canonical correlation analysis approach

**DOI:** 10.1007/s11357-024-01235-8

**Published:** 2024-06-18

**Authors:** Maud van Dinther, Paulien H. M. Voorter, Eleana Zhang, Sander M. J. van Kuijk, Jacobus F. A. Jansen, Robert J. van Oostenbrugge, Walter H. Backes, Julie Staals

**Affiliations:** 1 Department of Neurology, Maastricht University Medical Center, Maastricht, The Netherlands; 2 CARIM—School for Cardiovascular Diseases, Maastricht University, Maastricht, the Netherlands; 3 Department of Radiology and Nuclear Medicine, Maastricht University Medical Center, Maastricht, The Netherlands; 4 MHeNs—School for Mental Health and Neuroscience, Maastricht University, Maastricht, the Netherlands; 5 Department of Epidemiology and Medical Technology Assessment (KEMTA), Maastricht University, Maastricht, the Netherlands

**Keywords:** Cerebral small vessel disease, Neurovascular unit, Microvascular function, Cognitive function, Magnetic resonance imaging

## Abstract

Growing evidence indicates an important role of neurovascular unit (NVU) dysfunction in the pathophysiology of cerebral small vessel disease (cSVD). Individually measurable functions of the NVU have been correlated with cognitive function, but a combined analysis is lacking. We aimed to perform a unified analysis of NVU function and its relation with cognitive performance. The relationship between NVU function in the white matter and cognitive performance (both latent variables composed of multiple measurable variables) was investigated in 73 patients with cSVD (mean age 70 ± 10 years, 41% women) using canonical correlation analysis. MRI-based NVU function measures included (1) the intravoxel incoherent motion derived perfusion volume fraction (*f)* and microvascular diffusivity (D*), reflecting cerebral microvascular flow; (2) the IVIM derived intermediate volume fraction (*f*_*int*_), indicative of the perivascular clearance system; and (3) the dynamic contrast-enhanced MRI derived blood–brain barrier (BBB) leakage rate (*K*_*i*_) and leakage volume fraction (*V*_*L*_), reflecting BBB integrity. Cognitive performance was composed of 13 cognitive test scores. Canonical correlation analysis revealed a strong correlation between the latent variables NVU function and cognitive performance (*r* 0.73; *p* = 0.02). For the NVU, the dominating variables were D*, *f*_*int*_, and *K*_*i*_. Cognitive performance was driven by multiple cognitive tests comprising different cognitive domains. The functionality of the NVU is correlated with cognitive performance in cSVD. Instead of focusing on individual pathophysiological mechanisms, future studies should target NVU dysfunction as a whole to acquire a coherent understanding of the complex disease mechanisms that occur in the NVU in cSVD.

Trial registration: NTR3786 (Dutch Trial Register).

**Supplementary Information:**

The online version contains supplementary material available at 10.1007/s11357-024-01235-8.

## Introduction

Cerebral small vessel disease (cSVD) affects the small arteries, perforating arterioles, capillaries, and venules of the brain. Various pathological subtypes can be distinguished, with the deep perforator arteriopathy (or arteriosclerosis) being the most frequent type of cSVD. This type of cSVD is strongly associated with ageing, hypertension, and diabetes mellitus [1]. cSVD is characterized by neuroimaging markers such as white matter hyperintensities (WMHs) [1]. It may lead to lacunar stroke and is recognized as the leading cause of vascular cognitive impairment (VCI) [1]. Yet the underlying pathophysiological mechanisms are not fully understood. Growing evidence indicates an important role of the neurovascular unit (NVU) in the pathophysiology of cSVD [2].

**Fig. 1:**
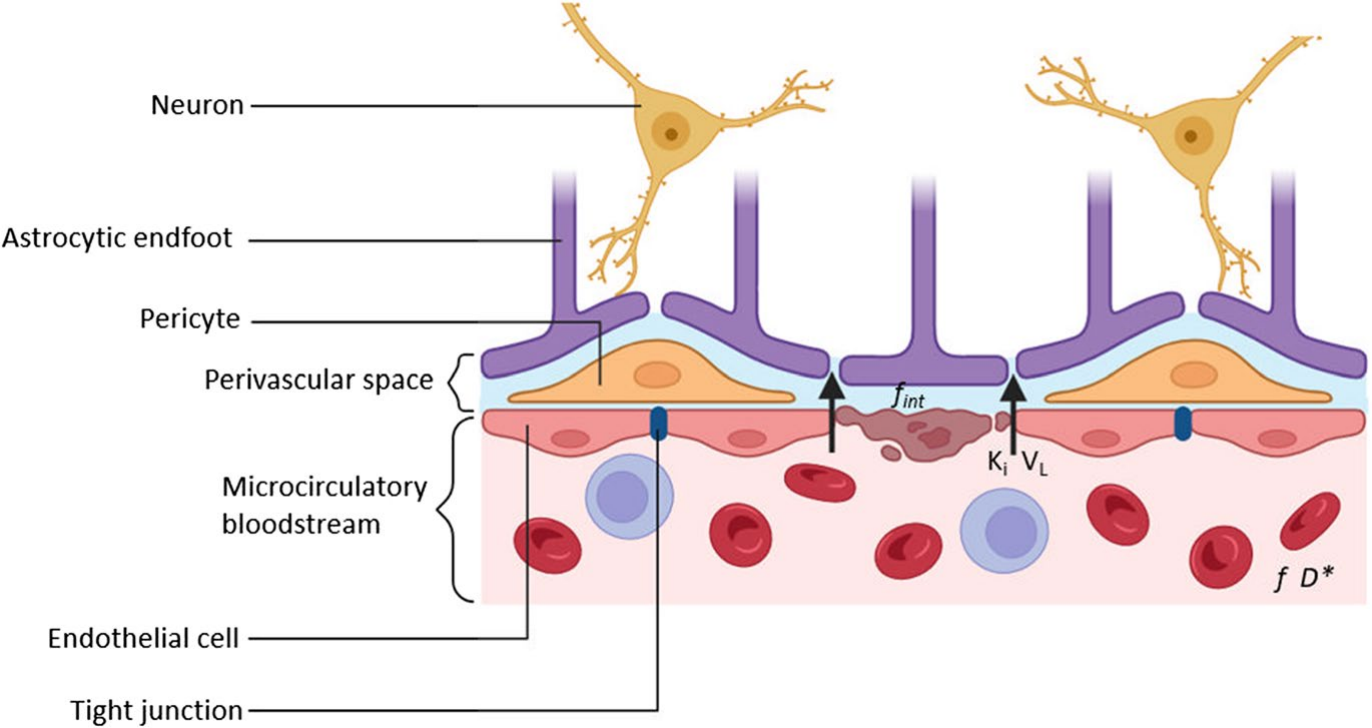
Schematic overview of neurovascular unit function measures derived from intravoxel incoherent motion and dynamic contrast-enhanced MRI. Intravoxel incoherent motion MRI provides several measures, including (1) the perfusion volume fraction (*f*), representing the voxel volume fraction of blood flowing through the capillaries, (2) the microvascular diffusivity (D*), proportional to the microcirculatory blood velocity, and (3) the intermediate volume fraction (*f*_*int*_), representing the interstitial fluid either between the parenchymal cells or within perivascular spaces. Using dynamic contrast-enhanced MRI, the blood–brain barrier leakage rate (*K*_*i*_) and leakage volume (*V*_*L*_) can be measured. *f*_*int*_ indicates the internediate volume fraction; *K*_*i*_, blood–brain barrier leakage rate; *V*_*L*_, blood–brain barrier leakage volume; *f*, perfusion volume fraction; D*, microvascular diffusivity. Image was created using Bio Render

The NVU is a functional–anatomical entity that represents the interplay between neurons and their feeding blood vessels, and is composed of endothelial cells, the basal membrane, pericytes, astrocytes, neurons, and the extracellular matrix around the vessels [3]. The NVU regulates cerebral blood flow (CBF) and perivascular clearance pathways, maintains the function of the blood–brain barrier (BBB), and is involved in the immune surveillance of the brain [4]. Using advanced physiological magnetic resonance imaging (MRI) techniques, such as intravoxel incoherent motion (IVIM) and dynamic contrast-enhanced (DCE) MRI, a proxy of several of these NVU functions can be measured in vivo (Fig. 1) [5]. The relation between several individual measurable functions of the NVU and cognitive function in CSVD has quite extensively been studied before [6–10]. However, previous studies lacked a common view of the underlying NVU, and its dysfunction as a whole. Moreover, the relative importance of the different pathophysiological processes occurring in the NVU remains unclear.

In this proof of concept study, we present a comprehensive approach to assess NVU function and its relation with cognitive function in patients with cSVD, by applying canonical correlation analysis (CCA) [11]. CCA is designed to find relationships between two latent concepts, represented by multivariable datasets (in our study: NVU function variables and cognitive test scores), thereby taking into account the interrelationship within a set of variables (i.e., different NVU function measures related to each other, as well as cognitive test scores related to each other) and between sets of variables (i.e., NVU function related to cognition) in a single model [12, 13]. The aim of this study is to explore whether the integrated functionality of the NVU, rather than separate functions of the NVU, is related to cognitive performance in patients with cSVD, in a comprehensive way.

## Material and methods

### Study population

This study uses previously collected data from a cross-sectional imaging study on cSVD [14]. We included patients with clinically manifest cSVD, consisting of patients with lacunar stroke or mild vascular cognitive impairment (mVCI) [14]. Lacunar stroke was defined as an acute lacunar stroke syndrome with a compatible recent small subcortical infarct on clinical brain MRI. If no such lesion was visible on MRI, or if no acute clinical brain MRI scan was performed, established clinical criteria for lacunar stroke syndrome were used [15, 16]. Stroke patients were included at least 3 months post-stroke to avoid acute stroke phase changes. Inclusion criteria for mVCI consisted of subjective cognitive complaints, objective cognitive impairment in at least one cognitive domain determined by neuropsychological assessment, and vascular lesions on brain MRI that suggested a link between the cognitive deficit and cSVD: moderate to severe WMHs (Fazekas score deep > 1 and/or periventricular > 2) or mild WMH (Fazekas score deep = 1 and/or periventricular = 2) combined with lacune(s) or microbleeds [17, 18]. Exclusion criteria included a suspicion of a neurodegenerative disease (e.g., Alzheimer’s disease), severe cognitive impairment (defined as a clinical dementia rating score > 1 or a Mini-Mental State Examination (MMSE) score of < 20), and other central nervous system diseases or contraindications for MRI. Lacunar stroke patients were also excluded in case of a symptomatic carotid stenosis of ≥ 50% or a possible cardioembolic source (e.g., atrial fibrillation). Participants were recruited from the Maastricht University Medical Centre and Zuyderland Medical Centre, the Netherlands. The study has been approved by the Medical Ethics Committee of the Maastricht University Medical Centre (Dutch Trial Register; NTR3786). All participants gave written informed consent.

### General and health characteristics

The presence of cardiovascular risk factors (i.e., hypertension, diabetes mellitus, hypercholesterolemia, smoking) was obtained from self-reported medical history and medication use. Body mass index (BMI) was calculated as weight in kilograms divided by height in meter squared. Educational level was registered and categorized based on the Dutch classification system of Verhage [19].

### Brain MRI acquisition

All patients underwent brain imaging on a 3-T MRI scanner (Achieva TX, Philips Healthcare, Best, The Netherlands) using a 32-element head coil suitable for parallel imaging. For anatomical segmentation a T1-weighted sequence (TR/TI/TE = 8.3/800/3.8 ms; field of view (FOV) 256 × 256 × 160 mm^3^; 1.0-mm cubic voxel size) and a T2-weighted fluid-attenuated inversion recovery (FLAIR) sequence (TR/TI/TE = 4800/1650/299 ms; FOV 256 × 256 × 180 mm^3^; 1.0-mm cubic voxel size) were performed.

DCE-MRI, providing measures of BBB integrity, was performed as described before [20]. Briefly, we used a dual-time resolution DCE-MRI to capture the fast signal changes during bolus arrival (fast sequence) and accurately measure the whole-brain tissue signal changes (slow sequence). Pre-contrast scans of both the fast and slow sequences were acquired, and a quantitative pre-contrast T1 relaxation time map was calculated to convert the contrast-enhanced signal intensities to concentrations in tissue. Subsequently, contrast agent (gadobutrol; dose 0.1 mmol/kg body weight) was injected.

IVIM imaging, providing measures of microvascular perfusion and the perivascular clearance system, was performed as described before [14]. In brief, a Stejskal-Tanner diffusion weighted (DW) spin echo single shot echo planar imaging pulse sequence (diffusion sensitization in anterior–posterior direction, *b* values 0, 5, 7, 10, 15, 20, 30, 40, 50, 60, 100, 200, 400, 700, and 1000 s/mm^2^) was used, which included an inversion recovery pulse (TI = 2230 ms) to suppress the cerebrospinal fluid.

### MRI analysis

#### Brain segmentation

Grey and white matter were automatically segmented on T1-weighted images (Freesurfer software) [21]. WMHs were automatically segmented on FLAIR images [22] and manually corrected by a trained investigator, and infarcts were excluded. For the current study, we restricted our main analysis to physiological MRI measures in the normal appearing white matter (NAWM), as the NAWM is regarded as ‘tissue at risk’, and multiple previous studies have shown early pathophysiological alterations in the NAWM in patients with cSVD [23–25]. As a secondary analysis, we also investigated physiological MRI measures in WMH.

#### DCE-MRI analysis

Analysis of the DCE-MRI data consisted of pharmacokinetic modelling and subsequent histogram analysis, as described previously [26]. The contrast agent concentration in tissue was calculated by using the relative signal enhancement and the quantitative T1 maps, and the vascular input function was derived from the superior sagittal sinus [27]. The graphical Patlak method was applied to the tissue concentration curves over time in a voxel-wise manner to derive the BBB leakage rate *K*_*i*_ (min^−1^) maps. The mean *K*_*i*_ of the NAWM and WMH was calculated. Additionally, the leakage volume (*V*_*L*_) was calculated using the histogram method as described before [23], representing the fractional volume of BBB leaking tissue that can be detected (i.e., the spatial extent of leakage). As *K*_*i*_ and *V*_*L*_ provide complementary information on BBB permeability, both variables were included in the CCA model as (inverse) measures of the NVU function ‘regulation of BBB integrity’.

#### IVIM MRI analysis

Pre-processing of DW images consisted of corrections for head displacements and spatial distortion (echo planar imaging and eddy current distortions) (FSL) [14]. A three-component IVIM (3C-IVIM) model was assumed, which describes the effects of the microcirculation, interstitial and perivascular fluid, and microstructural integrity on the DW signal. This 3C-IVIM model was fitted to the DW signal decay curves in a voxel-wise manner using the state-of-the-art physics-informed neural network fitting approach as described recently [28]. Although altered microstructural integrity may be a consequence of diminished NVU function, it does not provide a direct measure of NVU function and is therefore further disregarded. The extracted NVU function measures of interest included the microvascular diffusivity (D*), along with its corresponding microvascular perfusion volume fraction (*f*) and the intermediate volume fraction (*f*_*int*_). These 3C-IVIM measures were averaged within the NAWM and WMH. The D* reflects the fast directional changes in the microcirculatory bloodstream and depends on the microvascular blood velocity and the architecture of the microvascular bed. The *f* represents the volume of blood flowing through the capillaries. The microcirculation-related parameters D* and *f* were included in the CCA model as measures of the NVU function ‘regulation of CBF’. The *f*_*int*_ represents the interstitial fluid either between the parenchymal cells or within perivascular spaces, and is proposed to be indicative of the perivascular clearance system [29]. *f*_*int*_ was therefore included in the CCA analysis as an (inverse) measure of the NVU function ‘regulation of perivascular clearance pathways’.

### Neuropsychological assessment

We performed an extensive neuropsychological assessment covering three main cognitive domains: memory, executive function, and information processing speed. To examine memory, we performed the Rey Auditory Verbal Learning Test (RAVLT) (immediate recall, delayed recall, and delayed recognition) and the Digit Span Forward (subtest of Wechsler Adult Intelligence Scale (WAIS)-III) [30, 31]. To assess executive function, we used the Stroop Color-Word Test (SCWT) interference score (time of part 3 minus mean time of parts 1 and 2), Trail Making Test (TMT) interference score (time of part B minus time of part A), Category (animals and professions) and Letter Fluency, Letter-Number Sequencing (subtest of WAIS-III), and Digit Span Backward (subtest of WAIS-III) [31–35]. For examination of information processing speed, we performed the Symbol Substitution-Coding (subtest of WAIS-III), TMT part A, and SCWT parts 1 and 2 [31–33]. The scores of tests with higher scores representing worse performance (i.e., SCWT and TMT) were inverted, to simplify interpretation. All neuropsychological test scores were transformed into sample-based *z* scores (by dividing the difference between the individual raw test score and the overall group sample mean by the overall group sample standard deviation). For additional analysis, cognitive domain compound scores were determined by averaging the *z* scores of all tests within one domain. When one test score was missing, compound scores were calculated from the scores of the remaining tasks. In two patients, more than one test score in the executive domain was missing; for these patients, no reliable domain score could be calculated.

### Canonical correlation analysis

#### General concepts of CCA

CCA identifies sources of common variation and aims to correlate two multivariable datasets. CCA finds linear combinations of the variables of each set, such that the correlation between the linear combination of the one set and the linear combination of the other set is maximal. This results in multiple, uncorrelated, canonical modes. The number of canonical modes that is extracted is equal to the minimal number of variables in either of the variable sets [12].

The linear combination of the variables within one set, or the (latent) canonical variate, is computed from the weighted sum of the original variables as indicated by the canonical weight. Canonical loadings represent the correlation between a variable and its corresponding canonical variate. Canonical loadings are used for the interpretation of the nature of the relationship and explaining underlying constructs [36, 37]. Moreover, signs of the canonical loadings yield directional information about the variables’ contributions to the canonical relationship.

The canonical correlation is the Pearson correlation between the canonical variates of each set. Canonical correlations were squared to compute the proportion of variance shared by the linear composites of the two variable sets (the latent canonical variates). Using the canonical loadings, we computed the proportion of variance in the variable set which is extracted by its canonical variate. Additionally, a measure of redundancy was computed, which reflects how redundant one set of variables is, given the other set of variables. It provides a measure of the ability of a set of variables to explain variation in the other set of variables [37].

#### CCA for NVU function and cognitive performance

In this study, we investigated the correlation between NVU function in the NAWM and cognitive performance (both latent variates, composed of a set of measurable variables). NVU function variables (*K*_*i*_, *V*_*L*_, *f*, D*, and *f*_*int*_) were fed to the CCA model along with the 13 cognitive test scores. All variables were standardized by *z* score transformation (*z* = (subject value – population mean) / population SD). Five canonical modes were extracted (equal to the minimal number of variables within either of the variable sets; in this case NVU variables), and for each estimated mode, *p* values were calculated using random permutation testing (Wilk’s lambda, 1000 permutations). For each statistically significant mode, we obtained the Pearson correlation, the proportion of shared variance between the two latent canonical variates, and the variables’ canonical loadings. The proportion of the variables’ explained variance by its canonical variate and the redundancy were also obtained.

#### Additional analyses

Additional analyses were performed to assess result robustness. Firstly, CCA was repeated with all variables individually adjusted for age, sex, and educational level, and additionally for relative brain volume and relative WMH volume, using linear regression, to determine whether this had any impact on the results. Next, CCA was repeated with 3 cognitive domain *z* scores instead of 13 individual test scores to evaluate whether results remained similar.

As a secondary analysis, we investigated the correlation between NVU function in WMH and cognitive performance. Analyses were performed with SPSS software (v28.0; IBM, Chicago) and R (v4.3.3; R Foundation for Statistical Computing, Vienna). A *p* value lower than 0.05 was considered statistically significant.

## Results

### Characteristics of the study population

In the original study, 80 patients with clinically overt cSVD (44 with lacunar stroke and 36 with mVCI) were recruited [20]. Participants having image artefacts and image processing complications (*n* = 7) were excluded, resulting in 73 patients (40 with lacunar stroke and 33 with mild VCI) who were included in the current analyses. Table 1 presents the general characteristics of the study population. Descriptive statistics on NVU variables and cognitive test scores are shown in Table 2.

**Table 1:** Demographics and clinical characteristics of the study population

Characteristic	Study population (*N* = 73)
Age (years)	70.2 ± 10.8
Sex [*n*, (% women)]	30 (41.1)
Hypertension [*n*, (%)]	46 (63.0)
Diabetes mellitus [*n*, (%)]	12 (16.4)
Hypercholesterolemia [*n*, (%)]	42 (57.5)
Smoking [*n*, (%)]	18 (24.7)
BMI (kg/m^2^)	25.4 ± 3.9
Education, score	4.5 [4.0–5.0]
WMH volume (ml)	13.4 [7.1–30.5]

Data are presented as means ± standard deviation, median [interquartile range] or number of participants (percentages)

*BMI* body mass index, *WMH* white matter hyperintensity

**Table 2:** Neurovascular unit and cognitive function measures

Variable	Study population (*N* = 73)
NVU measures	
NAWM	
BBB leakage rate (*K*_*i*_) (10^–4^ min^−1^)	3.3 ± 1.6
BBB leakage volume (*V*_*L*_) (%)	35.7 ± 16.5
Perfusion volume fraction (*f*) (%)	1.1 ± 0.1
Microvascular diffusivity (D*) (10^–2^ mm^2^/s)	10.0 ± 0.1
Intermediate volume fraction (*f*_*int*_) (%)	9.3 ± 0.4
WMH	
BBB leakage rate (*K*_*i*_) (10^–4^ min^−1^)	3.6 ± 2.0
BBB leakage volume (*V*_*L*_) (%)	42.0 ± 20.1
Perfusion volume fraction (*f*) (%)	1.6 ± 0.2
Microvascular diffusivity (D*) (10^–2^ mm^2^/s)	9.4 ± 0.3
Intermediate volume fraction (*f*_*int*_) (%)	17.7 ± 2.9
Cognitive test scores	
Memory	
RAVLT immediate recall	32.1 ± 10.3
RAVLT delayed recall	5.4 ± 3.5
RAVLT delayed recognition	11.4 ± 3.2
Digit span forward	7.4 ± 2.0
Executive function	
TMT interference score	118.5 ± 100.8
SCWT interference score	87.5 ± 53.0
Fluency categories	30.3 ± 10.6
Fluency letters	24.8 ± 12.6
Letter-number sequencing	6.7 ± 3.3
Digit span backwards	5.3 ± 1.7
Psychomotor speed	
TMT A (s)	66.1 ± 34.5
SCWT I (s)	57.8 ± 14.8
SCWT II (s)	76.3 ± 20.1
Symbol-Substitution-Coding	42.5 ± 18.7

Data are presented as means ± standard deviation

*NVU* neurovascular unit, *BBB* blood–brain barrier, *NAWM* normal appearing white matter, *RAVLT* Ray Auditory Verbal Learning Test, *TMT* Trail Making Test, *SCWT* Stroop Color-Word Test

### CCA for NVU function and cognitive performance

**Fig. 2:**
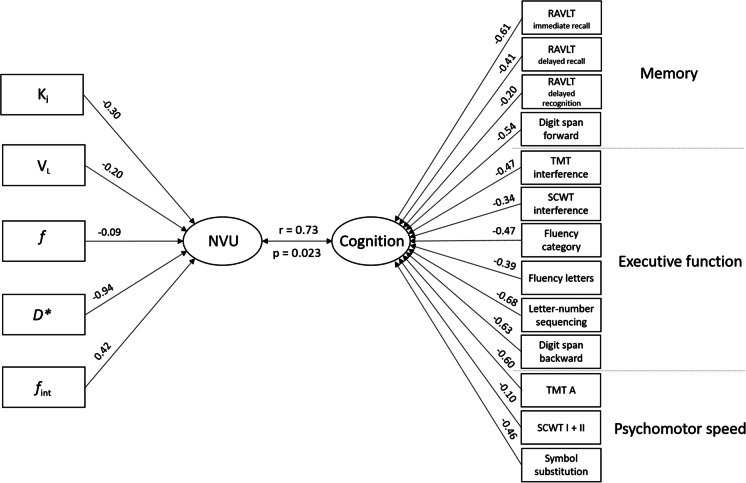
Canonical correlation analysis of the neurovascular unit and cognitive function. Canonical loadings (correlation between variables and the corresponding latent canonical variate) and the canonical correlation (correlation between two latent canonical variates) are presented. Scores of tests with higher scores representing worse performance (i.e. TMT and SCWT) were inverted before they were fed into the CCA model. *K*_*i*_ indicates leakage rate; *V*_*L*_, leakage volume; *f*, perfusion volume fraction; D*, microvascular diffusivity; *f*_*int*_, internediate volume fraction; RAVLT, Ray Auditory Verbal Learning Test; TMT, Trail Making Test; SCWT, Stroop Color-Word Test

After entering the five NVU variables of the NAWM and all 13 cognitive test scores in the CCA model, five canonical modes were extracted. We identified a single statistically significant mode (*p* = 0.023), with an (unadjusted) canonical correlation of 0.73 between the two canonical variates. The shared common variance between the canonical variates was 53.3%. A schematic overview of CCA results, including the canonical loadings for each variable, is visualized in Fig. 2. The NVU function canonical variate was mainly driven by D*, *f*_int_, and *K*_*i*_, while the cognitive function variate was driven by various cognitive tests compromising multiple cognitive domains. Results of the remaining four non-significant modes can be found in Supplementary Table S1 and S2. Cross-correlation results between all individual variables included in the CCA analysis are depicted in Supplementary Figure S1.

The latent NVU function canonical variate, on average, explained 24.0% of variance in the measured NVU function variables. The latent cognition canonical variate, on average, explained 23.1% of the variance in all cognitive test scores. The total redundancy of the 5 extracted canonical modes in the NVU set (given the cognition set) was 26.1%, with the one significant mode accounting for 12.8% of this redundancy. The total redundancy in the cognition set (given the NVU set) was 18.7%, with the one significant mode accounting for 12.3% of this redundancy.

### Additional analyses

When CCA was repeated with all variables individually adjusted for age, sex, and educational level, results remained essentially the same. A single significant canonical mode was extracted (*p* = 0.048), with a canonical correlation of 0.72 between canonical variates. A schematic overview of these results, including the canonical loadings for each variable, can be visualised in Supplementary Figure S2. In this adjusted model, NVU function was mainly driven by D* and *K*_*i*_, and cognitive function was driven by multiple cognitive tests compromising multiple cognitive domains. When next to age, sex, and educational level, an additional adjustment for relative brain volume and relative WMH volume was added; results remained similar (Supplementary Figure S3; canonical correlation 0.71, *p* = 0.046, similar canonical loadings for all variables).

When we substituted the individual test scores with three cognitive domain *z* scores for memory, executive function, and psychomotor speed, similar results were obtained (Supplementary Figure S4). The canonical correlation between the canonical variates was 0.58 for the single significant canonical mode (*p* = 0.001). Again, NVU function was mainly driven by D* and *K*_*i*_, although in this model, all NVU variables seemed to be relevant drivers of the canonical variate (i.e., approximately all canonical loadings ≥ 0.3). Again, all three cognitive domains had substantial canonical loadings.

When CCA was repeated with NVU function variables in WMH instead of NAWM, we did not find any statically significant mode (first canonical mode: *p* = 0.066).

## Discussion

In this proof-of-concept study, we applied CCA to test a comprehensive approach of NVU dysfunction in cSVD. While previous studies showed associations with individual features occurring in the NVU [6–10], we showed that the NVU, as a whole functionary unit, is related to cognitive function in patients with cSVD.

The NVU regulates multiple physiological processes including CBF, perivascular clearance pathways, BBB permeability, and the immune defence. In cSVD, the pathophysiological mechanisms that are hypothesized to occur in the NVU are complex. It has previously been suggested that a defect in one functional component of the NVU can affect other NVU components. As such, lower CBF results in lower shear stress, which can lead to lower expression of tight junctions and hence a more permeable BBB [38]. Lower CBF can also cause hypoxia, which induces an inflammatory response, subsequently also affecting tight junctions and leading to stronger BBB permeability [39, 40]. Vice versa, BBB disruption can induce neuroinflammation as well, by releasing inflammatory mediators and by enhancing peripheral-central immune response crosstalk [41, 42]. Subsequently, inflammation and BBB impairment both also have an effect on perivascular clearance pathways. Inflammation directly slows down convective flow and impairs perivascular clearance, and a more permeable BBB leads to leakage of fluids and molecules, which can block interstitial fluid flow in the perivascular spaces [42]. In turn, the buildup of toxic substances due to impaired perivascular clearance can cause further damage to the BBB [42]. Although this overview is far from complete, it is clear that impairment in one of the NVU functions can result in a cascade of events that leads to dysregulation of other NVU functions, and subsequently ends in a vicious circle that results in deterioration of the NVU [39]. Eventually, this causes brain tissue injury and can lead to worsening of cognitive function [2].

The relationship between individual functions of the NVU and cognitive performance has been studied before. However, studies investigating cross-sectional associations between CBF, BBB permeability, and the perivascular clearance system, on the one hand, and cognitive function, on the other hand, have yielded inconsistent results [6, 8, 10, 43–45]. One could imagine that whether or not neuronal damage, and eventually cognitive dysfunction occurs, and to which extent, does not depend on a single NVU feature but on the interplay of different NVU components. Therefore, a combination of different NVU functions probably can predict cognitive function better than single NVU functions. We showed a quite strong correlation, and a substantial amount of shared variance, between the latent NVU function variable and cognitive performance in patients with cSVD in our study. As expected, the redundancy was lower than the shared variance that was estimated by correlating the optimal linear correlation, which is known to ‘inflate’ as it represents an optimum weighting of both sides of the relationship [37]. Nonetheless, the derived redundancy still indicates a substantial amount of shared variance.

A few former studies have investigated the mutual relationship between different NVU functions in human in vivo. As such, it has been demonstrated that stronger BBB leakage is linked with lower cerebral blood flow [23]. Furthermore, a positive correlation between BBB leakage and the number of enlarged perivascular spaces has been shown [46, 47], indicating that more BBB leakage is associated with dysfunction in the perivascular clearance system. Likewise, circulating inflammatory markers have also been associated with a higher number of enlarged perivascular spaces [48]. These results, linking different NVU functions with each other, support our approach to analyse the NVU as a whole functionary unit.

In our model, D* (proportional to the microcirculatory blood velocity), *f*_*int*_ (a proxy for perivascular clearance) and *K*_*i*_ (BBB leakage rate) were the main determinants of NVU function in NAWM. Hence, measures that reflect all different NVU functions that were included in the model did contribute to the canonical correlation. This supports the concomitant and reciprocal contribution of different NVU functions in the pathophysiology of cSVD and stresses the importance of a combined analysis of different NVU functions.

The directionality of the correlations between NVU function measures in the NAWM and cognitive function in the CCA model were as expected for most of the imaging parameters. As such, lower *f* and lower D*, both reflective of lower perfusion in the microcirculation, correlated with lower cognitive performance. Likewise, a higher *f*_*int*_, indicative of more perivascular and interstitial fluid, which could point at worse perivascular clearance, correlated with lower cognitive performance. However, the direction of the correlation between the BBB-related measures *K*_*i*_ and *V*_*L*_ and cognitive function was unexpected. As such, higher *K*_*i*_ and higher *V*_*L*_, both reflective of higher BBB leakage, correlated with higher cognitive performance, whereas one would expect higher BBB leakage to correlate with lower cognitive performance. Nonetheless, it has been argued before that the interpretation of *K*_*i*_ is not straightforward in the context of cSVD [49]. In case of subtle BBB leakage, as seen in cSVD, *K*_*i*_ approaches the product of the BBB permeability (*P*) and the blood vessel surface area (*S*) [49]. In cSVD, there is an increase in vessel wall permeability, whilst at the same time, the microvessel surface area is reduced due to (functional or structural) microvascular rarefaction [5], resulting in counterbalancing effects in *K*_*i*_. Hence, when the decrease in vessel density dominates over the increase in vessel permeability, a lower *K*_*i*_ and *V*_*L*_ can also be indicative of microvascular disease. This could explain the unexpected positive correlation between BBB leakage measures and cognitive function.

We confirmed a correlation between NVU function in the NAWM, but not NVU function in WMHs, and cognitive performance. This is not surprising, as WMHs reflect zones of tissue damage as a result of advanced microvascular disease, and within WMHs, the NVU is probably already far deteriorated. Besides, technical factors could also have played a role, as DCE-MRI and IVIM measures are sensitive to image noise. Where the effect of image noise of larger regions of interest such as the NAWM is averaged out, values calculated over small regions of interest, such as WMHs, are less reliable.

NVU function in the NAWM correlated with various cognitive test scores that comprised all different cognitive domains. This pattern was replicated in the additional model using cognitive domain scores instead of individual test scores, in which high canonical loadings were shown for all three cognitive domains. These findings are in line with a recent meta-analysis that showed that cSVD affects all major domains of cognitive ability [50].

The applied joint modelling of different NVU function measures in the current study exploits the existence of shared underlying processes between changes in perfusion, BBB leakage and dysfunction of perivascular clearance pathways. We have demonstrated the potential value of CCA as a novel approach to study NVU function, as it can describe many-variable-to-many-variable relations [12]. CCA overcomes the issues of loss of information and lack of sensitivity which can result from interpretation of parallel univariate analyses. Moreover, CCA takes into account the interrelationships within sets of variables, whereas typical regression analysis provides an estimate for the association between a given independent variable and the dependent variable holding all other variables constant. As different NVU functions are reciprocally related to each other, CCA probably is more suitable to study NVU function. However, our study is exploratory in nature and only provides a single application of this method using real-life data. Cross-validation studies are needed to determine the validity and robustness of CCA for a comprehensive NVU analysis. Moreover, different statistical approaches that can provide a combined analysis of different NVU functions might also be appropriate in studying the complex pathophysiology of cSVD.

Few limitations of the study need to be acknowledged. Firstly, our integrated model might not include all relevant NVU aspects. For example, the current model lacks a measure of inflammation, as validated inflammatory blood biomarkers were not available in our study population. Despite, we were still able to provide proof of concept for analysing the NVU as a whole functionary unit. Secondly, although our study demonstrates a relation between NVU function and cognitive performance, causality cannot be inferred. Thirdly, although previous studies have suggested that *f*_*int*_ can be used as a marker for the perivascular clearance system [29, 51], it remains to be investigated how accurately IVIM can detect clearance system failure. Yet, no other non-invasive physiological imaging techniques that can provide a proxy for perivascular clearance have been validated [49]. Notwithstanding these limitations, the strengths of our study include the novel approach of using CCA for a comprehensive analysis of NVU function, the multimodal MRI protocol providing us with several measures for NVU function, the use of an extensive cognitive test battery enabling us to examine a spectrum of cognitive domains and the well-represented spectrum of clinically overt cSVD patients with different stages of disease severity.

In conclusion, we demonstrated that the NVU, as a whole functionary unit, is related to cognitive performance in patients with cSVD. Our findings support the concomitant contribution of different NVU functions in the pathogenesis of cSVD. Instead of focussing on individual pathogenic mechanisms, we suggest for future studies in cSVD to consider the interaction between different processes and to target NVU function as a whole to acquire coherent understanding of the disease mechanisms in cSVD.

### Supplementary Information

Below is the link to the electronic supplementary material.

Supplementary file1 (DOCX 429 KB)

## Data Availability

On request. The online version contains supplementary material at *DOI*.
