# Supplementary material for: The neurovascular unit and its correlation with cognitive performance in patients with cerebral small vessel disease: a canonical correlation analysis approach
Source: GeroScience. 2024 Jun 18;46(5):5061–73. doi: 10.1007/s11357-024-01235-8 (PMC11335703; doi:10.1007/s11357-024-01235-8)
Supplement: Supplementary file 1 — Supplementary file1 (DOCX 429 KB) [file 11357_2024_1235_MOESM1_ESM.docx]

**Supplementary data**

**The neurovascular unit and its correlation with cognitive performance in patients with cerebral small vessel disease: a canonical correlation analysis approach**

Maud van Dinther^1,2^, Paulien H.M. Voorter^3,4^, Eleana Zhang^1^, Sander M.J. van Kuijk^5^, Jacobus F.A. Jansen^3,4^, Robert J. van Oostenbrugge^1,2,4^, Walter H. Backes^2,3,4^, Julie Staals^1,2^.

1. Department of Neurology, Maastricht University Medical Center, Maastricht, The Netherlands
2. CARIM - School for Cardiovascular Diseases, Maastricht University, Maastricht, the Netherlands
3. Department of Radiology and Nuclear Medicine, Maastricht University Medical Center, Maastricht, The Netherlands
4. MHeNs - School for Mental Health and Neuroscience, Maastricht University, Maastricht, the Netherlands
5. Department of Epidemiology and Medical Technology Assessment (KEMTA), Maastricht University, Maastricht, the Netherlands.

**Corresponding author:** Maud van Dinther, e-mail: [maud.van.dinther@mumc.nl](mailto:maud.van.dinther@mumc.nl), postal address: Postbus 5800, 6202 AZ Maastricht, The Netherlands.

**Table S1.** Results of canonical correlation analysis for NVU function and cognition performance

| **Mode** | **Canonical Correlation** | **Permutation based p-value** | **Parametric based p-value** |
| --- | --- | --- | --- |
| 1 | 0.73 | 0.023* | 0.019* |
| 2 | 0.54 | 0.085 | 0.409 |
| 3 | 0.50 | 0.059 | 0.570 |
| 4 | 0.45 | 0.064 | 0.762 |
| 5 | 0.22 | 0.652 | 0.976 |

* indicates a statically significant canonical correlation

**Table S2.** Canonical loadings for all modes from canonical correlation analysis for NVU function and cognition performance

|  | **Mode 1** | **Mode 2** | **Mode 3** | **Mode 4** | **Mode 5** |
| --- | --- | --- | --- | --- | --- |
| **NVU variables** |  |  |  |  |  |
| *K_i_* | -0.30 | -0.14 | -0.39 | 0.69 | 0.52 |
| *V_L_* | -0.20 | -0.15 | 0.19 | 0.81 | 0.49 |
| *f* | -0.09 | -0.75 | -0.12 | 0.32 | -0.56 |
| *D** | -0.94 | -0.13 | 0.24 | -0.02 | -0.19 |
| *f_int_* | 0.42 | -0.30 | -0.24 | 0.57 | -0.60 |
|  |  |  |  |  |  |
| **Cognition variables** |  |  |  |  |  |
| RAVLT, immediate recall | -0.61 | -0.09 | 0.09 | -0.21 | -0.32 |
| RAVLT, delayed recall | -0.41 | 0.01 | 0.24 | -0.38 | -0.21 |
| RAVLT, delayed recognition | -0.20 | -0.26 | -0.01 | -0.40 | -0.03 |
| Digit span forward | -0.54 | -0.17 | -0.14 | 0.48 | 0.27 |
| TMT interference | -0.47 | -0.35 | -0.40 | -0.23 | -0.17 |
| SCWT interference | -0.34 | -0.48 | 0.10 | 0.26 | -0.50 |
| Fluency, category | -0.47 | -0.41 | 0.04 | -0.03 | 0.32 |
| Fluency, letters | -0.39 | -0.35 | 0.01 | -0.23 | 0.24 |
| Letter-number sequencing | -0.68 | -0.18 | -0.04 | 0.10 | 0.03 |
| Digit span backward | -0.63 | -0.18 | -0.05 | 0.23 | 0.14 |
| TMT A | -0.60 | -0.41 | 0.46 | -0.28 | -0.10 |
| SCWT I + II | -0.10 | -0.76 | 0.22 | -0.02 | 0.18 |
| Symbol substitution | -0.46 | -0.22 | 0.27 | -0.15 | -0.02 |

Scores of tests with higher scores representing worse performance (i.e. TMT and SCWT) were inverted before they were fed into the CCA model. Abbreviations: K_i_ indicates leakage rate; V_L_, leakage volume; *f*, perfusion volume fraction; *D**, microvascular diffusivity; *f_int_*, intermediate volume fraction; RAVLT, Ray Auditory Verbal Learning Test; TMT, Trail Making Test; SCWT, Stroop Color-Word Test.


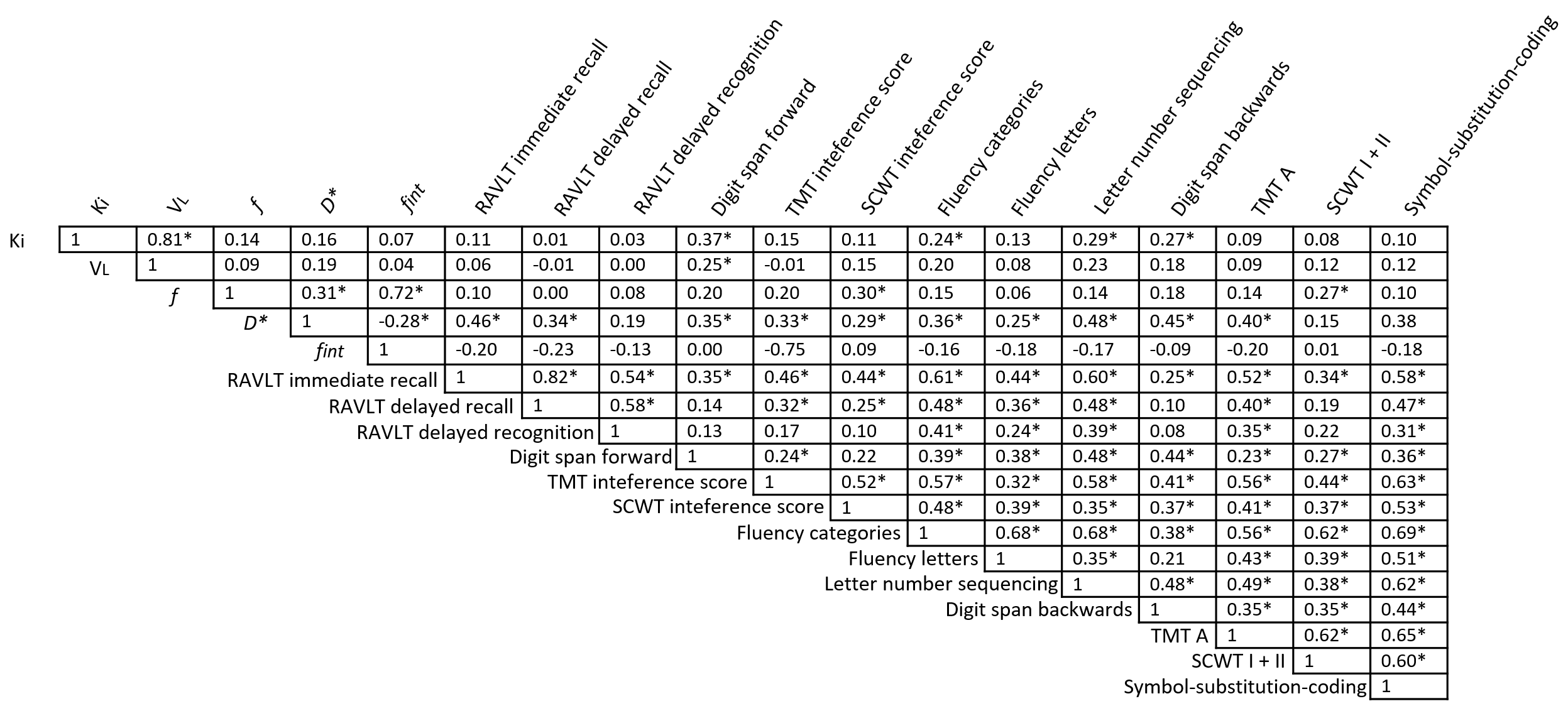
 **Fig. S1** Bivariate Pearson correlations of neurovascular unit variables and cognitive test scores
* indicates a statically significant correlation
Scores of tests with higher scores representing worse performance (i.e. TMT and SCWT) were inverted.
Abbreviations: Ki indicates leakage rate; VL, leakage volume; *f*, perfusion volume fraction; *D**, microvascular diffusivity; *fint*, intermediate volume fraction; RAVLT, Ray Auditory Verbal Learning Test; TMT, Trail Making Test; SCWT, Stroop Color-Word Test.


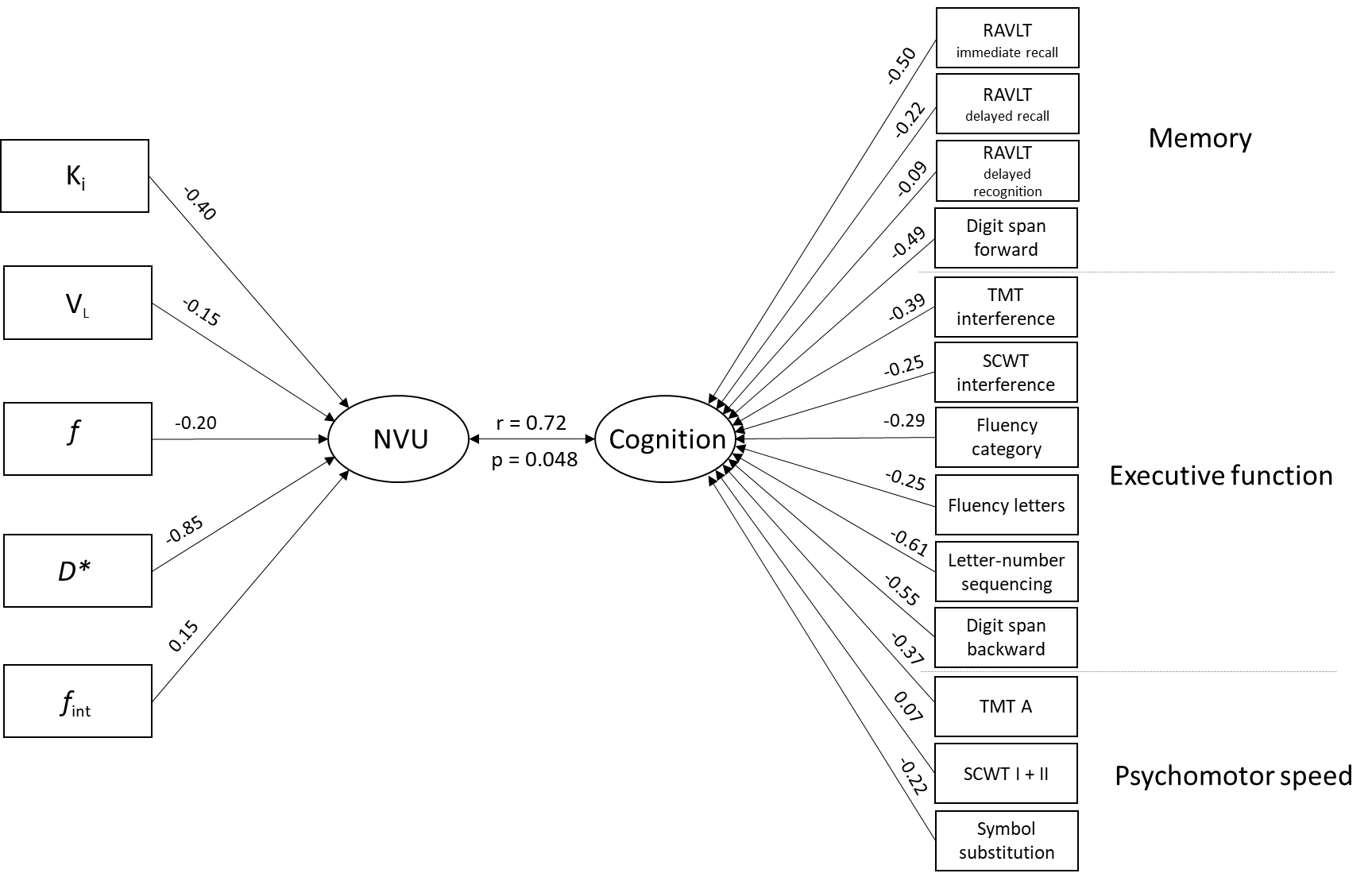
 **Fig. S2 Canonical correlation analysis of the NVU and cognitive function, adjusted for sex, age and educational level**
Canonical loadings (correlation between variables and the corresponding latent canonical variate) and the canonical correlation (correlation between two latent canonical variates) are presented.
Scores of tests with higher scores representing worse performance (i.e. TMT and SCWT) were inverted before they were fed into the CCA model. Abbreviations: K_i_ indicates leakage rate; V_L_, leakage volume; *f*, perfusion volume fraction; *D**, microvascular diffusivity; *f_int_*, intermediate volume fraction; RAVLT, Ray Auditory Verbal Learning Test; TMT, Trail Making Test; SCWT, Stroop Color-Word Test.

**
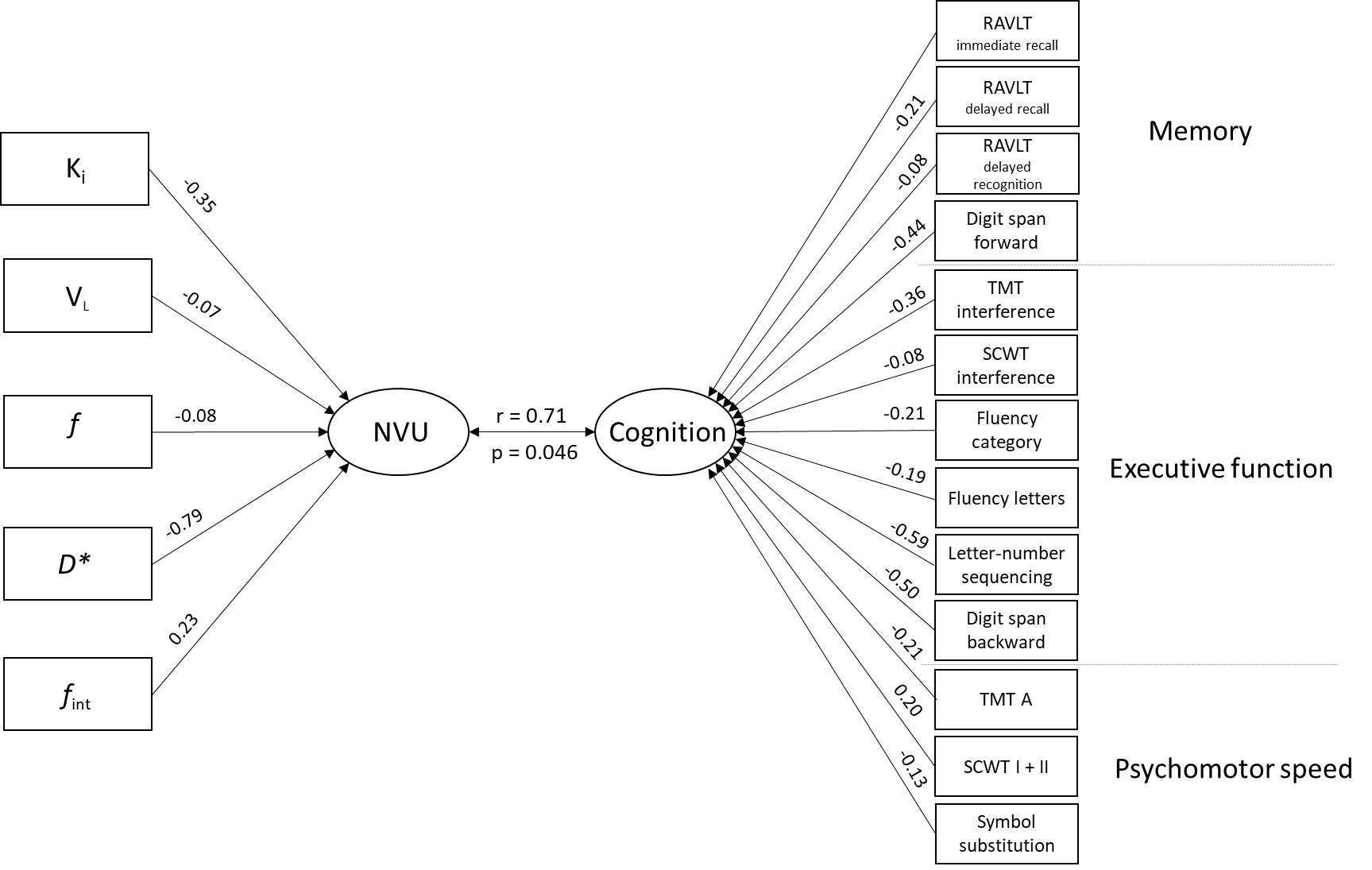

Fig. S3 Canonical correlation analysis of the NVU and cognitive function, adjusted for sex, age, educational level, relative brain volume and relative WMH volume**
Canonical loadings (correlation between variables and the corresponding latent canonical variate) and the canonical correlation (correlation between two latent canonical variates) are presented.
Scores of tests with higher scores representing worse performance (i.e. TMT and SCWT) were inverted before they were fed into the CCA model. Abbreviations: K_i_ indicates leakage rate; V_L_, leakage volume; *f*, perfusion volume fraction; *D**, microvascular diffusivity; *f_int_*, intermediate volume fraction; RAVLT, Ray Auditory Verbal Learning Test; TMT, Trail Making Test; SCWT, Stroop Color-Word Test.

**
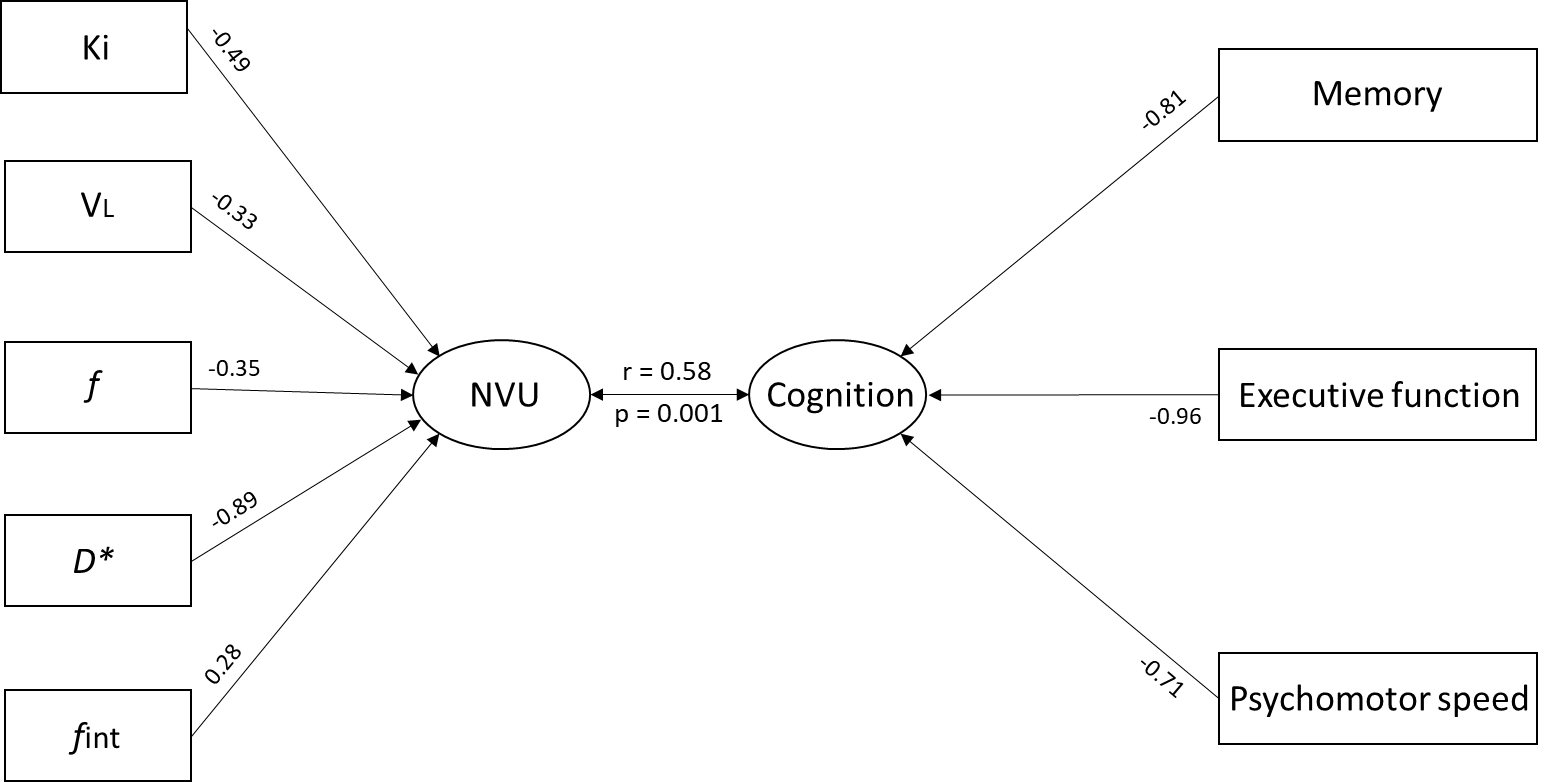

Fig. S4 Canonical correlation analysis of the NVU and cognitive function, in which cognitive function is represented by cognitive domains scores instead of individual test scores.**
Canonical loadings (correlation between variables and the corresponding latent canonical variate) and the canonical correlation (correlation between two latent canonical variates) are presented.
Abbreviations: K_i_ indicates leakage rate; V_L_, leakage volume; *f*, perfusion volume fraction; *D**, microvascular diffusivity; *f_int_*, intermediate volume fraction.
